# A Risk Profile of Sociodemographic Factors in the Onset of Academic Burnout Syndrome in a Sample of University Students

**DOI:** 10.3390/ijerph16050707

**Published:** 2019-02-27

**Authors:** Raimundo Aguayo, Gustavo R. Cañadas, Latifa Assbaa-Kaddouri, Guillermo A. Cañadas-De la Fuente, Lucía Ramírez-Baena, Elena Ortega-Campos

**Affiliations:** 1 Campus Universitario de Cartuja, Faculty of Psychology, University of Granada, 18071 Granada, Spain; raguayo@ugr.es; 2 Campus Universitario de la Cartuja, Department of Didactic of Mathematics, Faculty of Education, University of Granada, 18071 Granada, Spain; grcanadas@ugr.es; 3 Social Psychology Department, Faculty of Social Sciences, University of Granada, Santander, 1, 52005 Melilla, Spain; latifaassbaa@correo.ugr.es; 4 Nursing Department, Faculty of Health Sciences, University of Granada, Avenida de la Ilustración, 60, 18016 Granada, Spain; 5 Campus Universitario de Cartuja, Brain, Mind and Behaviour Research Center (CIMCYC), University of Granada, 18071 Granada, Spain; luciarb@correo.ugr.es; 6 Faculty of Psychology, University of Almería, Carretera de Sacramento s/n, 04120 Almería, Spain; elenaortega@ugr.es

**Keywords:** academic burnout syndrome, university students, prevalence, sociodemographic factors, stress

## Abstract

Studying for a university degree can be very demanding, as students must cope with a variety of academic, social and personal challenges. If these demands persist, and if there are insufficient resources with which to address them, they will eventually provoke stress. When stress is present for long periods of time, it can lead to academic burnout syndrome, the signs of which are emotional exhaustion, depersonalisation and inadequate personal accomplishment. This paper considers certain sociodemographic factors (age, sex, children, marital status, employment status, degree subject, faculty, academic year) in the identification of a risk profile of developing burnout syndrome. This study is cross-sectional, associative and ex post facto. The Maslach Burnout Inventory-Student Survey was administered to 445 students in the University of Granada. According to the risk profile obtained, first-year male students in Primary Education and Social Education courses are at risk of developing burnout syndrome.

## 1. Introduction

Studying for a university degree can be very demanding, generating considerable stress [1,2]. Academic demands are high, and students must cope with a variety of academic, social and personal challenges. Many hours of work must be dedicated to searching for information, tasks must be prepared and carried out and exams prepared for. In addition to academic obligations, other stresses are experienced, such as the change of address, living away from home, the search for new interpersonal relationships and increasing concerns with finding postgraduate employment in line with the degree obtained [2,3,4,5]. These demands, if they persist over time in the absence of sufficient resources to resolve them, may generate prolonged malaise and the development of burnout syndrome [3].

Traditionally, burnout has been studied with respect to persons in professions such as teaching, medicine and the police [6,7,8,9,10]. Recently, however, studies have considered other population groups, including informal caregivers, housewives and students [11,12]. In this respect, university students can be equated with employed persons, with whom they share many working conditions [2,5,13,14]. In both cases, there is a relationship with an organisation or institution to which services or products are offered, in return for direct or indirect compensation. Workers receive monetary incentives for accomplishing the goals set for them, while students achieve academic and social recognition.

Burnout syndrome is a psychological problem caused by a continual subjection to stress factors, which can make students feel unable to fulfil their responsibilities, lose interest in their studies and doubt their ability to achieve academic goals. The simultaneous presence of these manifestations is termed academic burnout syndrome [15], the most widely accepted definition of which was given by Maslach and Jackson [16], who referred to a three-dimensional structure: emotional exhaustion, depersonalisation or cynicism, and low personal accomplishment or inefficacy. Subsequently, other authors have adapted this proposal to the student population [17,18,19].

Emotional exhaustion (EE) describes a sensation of a lack of energy and the depletion of emotional resources. Depersonalisation (D) refers to feelings of estrangement from studies, together with indifferent and cynical attitudes towards peers and teachers. Low levels of personal accomplishment (PA) refers to perceptions of inefficacy and a lack of competence in the performance of university tasks.

Empirical studies have shown that the prevalence of academic burnout varies considerably depending on the university degree in question. In this respect, Barboza and Beresin [20] concluded that 73.5% of nursing students experienced little sense of personal accomplishment; Caballero, Abello and Palacio [21] observed academic burnout in 41.6% of psychology students; and Bittar [22] reported the following prevalences of burnout, by university degree subject: 34% in law, 13% in psychology and public administration, 10% in business studies, 9% in accounting, 8% in international relations, 6% in information science, 3% in tourism and 2% in communication.

The variables related to academic burnout are very diverse, encompassing sociodemographic and psychological factors as well as those related to the university context. A study of these sociodemographic characteristics might produce a risk profile of those vulnerable to burnout syndrome. Among these variables, those which have aroused most interest to date are age and sex. Nevertheless, neither age [23] nor sex [2,5,14,24] seem to play any significant role in the onset of burnout. The university degree studied has also been considered a variable of research interest with respect to the prevalence of burnout, but disparate results have been reported.

Academic burnout can have serious physical and psychological consequences for students’ health [13,15,25,26]. These consequences may be psychosomatic (e.g., cardiovascular problems, gastrointestinal disorders, lack of sleep, fatigue), emotional (e.g., dissatisfaction with studies, depression, lack of self-esteem, demotivation) or behavioural (e.g., low academic performance, alcohol and/or drug abuse, poor diet, absenteeism, dropout).

This paper seeks to clarify certain aspects of academic burnout syndrome in a sample of Spanish university students in different academic fields (education, nursing and social sciences). The specific goals are: (a) to analyse the relationship between the syndrome and certain sociodemographic risk factors (age, sex, marital status, employment status, degree subject, faculty and academic year); and (b) to elaborate a risk profile regarding the above factors. To our knowledge, no previous research has addressed the latter goal, and so we believe the present paper makes a useful contribution to the literature, highlighting the need to identify a burnout risk profile for university students and providing initial results in this respect.

## 2. Materials and Methods

### 2.1. Sample

The sole criterion for inclusion in this study was that the student should be enrolled in a degree subject at the Melilla campus of the University of Granada. The students in the study sample were recruited from the three faculties of this university campus, and selected by non-probabilistic sampling by quotas (the stratification variable was the faculty). The response rate was 100%, and a total of 445 university students participated voluntarily in the study. For a 5% sampling error, the minimum number of participants required was 405 [27]. The sample was distributed as follows: 118 students from the Faculty of Nursing (26.5%), 193 from the Faculty of Education and Humanities (43.4%) and 134 from the Faculty of Social Sciences (30.1%). By academic years, 189 (42.5%) were first-year students, 130 (29.2%) were in their second year, 98 (22%) in the third year and 28 (0.6%) in the fourth.

The ages of the students ranged from 18 to 50 years, with a mean of 21.85 years (SD = 4.46). Of the total sample, 278 were women (62.5%). With respect to marital status and children, 95.3% had no spouse or children. In relation to paid employment, 88.3% of the students did not have a job.

### 2.2. Study Instruments

The study data regarding age, sex, marital status, children, employment, faculty, degree subject and academic year were obtained using the MBI-Student Survey [28], an ad hoc sociodemographic data questionnaire. This instrument is composed of 16 items, addressing the three dimensions of the syndrome, scored on a 7-point response scale (Never = 0; A few times a year = 1; Once a month at most = 2; A few times a month = 3; Once a week = 4; A few times a week = 5; Every day = 6). In the sample, the Cronbach’s alpha statistic was 0.78 for EE, 0.63 for D and 0.81 for PA.

### 2.3. Procedure and Data Analysis

The study was conducted according to a cross-sectional, associative, ex post facto design [29]. The data were collected at the three faculties of the Melilla campus in June 2017. The questionnaires were distributed over a two-week period and data collection was carried out in the classrooms during class time, with the approval of the deans and teachers involved. The students gave their informed consent to participate, and confidentiality and anonymity at all times were assured.

Descriptive statistics were calculated for all variables. For the first goal, differences in the qualitative independent variables were analysed by Student’s t test for the dichotomous variables and by ANOVA for the polytomous ones, and the presence of normal distribution and homoscedasticity was confirmed by the Kolmogorov-Smirnov test and by Levene’s test, respectively. The post hoc analyses were performed using the Tukey statistics, since homogeneity of the variances could be assumed. The effect size used in these analyses was Cohen’s d. Pearson’s product-moment bivariate correlation was used to determine associations between continuous variables.

For the second goal, two techniques were used in order to elaborate a risk profile of sociodemographic factors. First, a two-step cluster analysis (TSCA) was performed to identify patterns in the responses of these variables. This method of analysis was selected because of the large sample size and the presence of qualitative categorical variables. TSCA determines the importance and ranking of categorical variables which play a role in predicting the model, and obtains the number of clusters automatically. The fitness of the model was determined using Schwarz’s Bayesian Information Criterion (BIC) using the average silhouette coefficient. The similarity measure was calculated with the log-likelihood method. A one-way ANOVA with the burnout dimensions as dependent variables and cluster assignment as the independent variable was also computed. The second technique used was that of multiple linear regression models. These were derived using the backwards stepwise method, due to the lack of theoretical and empirical agreement on the relevance of these variables in relation to the onset of academic burnout syndrome [30]. In this procedure, the predictors are entered into the model based on a mathematical criterion [30,31]. Only the variables that were significant in the univariate analyses were included in these models, following the recommended guidelines for balance between the sample size and the number of predictors [30,31,32]. The assumptions of normality, linearity, homoscedasticity, multicollinearity and independence of errors were verified. The proportion of missing data was less than 5%, and this question was addressed using pairwise deletion. All statistical analyses were performed with IBM SPSS 23.0 and R 3.3.1.

### 2.4. Ethics

This study has been approved by the Ethics Committee of the University (ethical code number 393/CEIH2017) and has therefore been performed in accordance with the ethical standards laid down in an appropriate version of the Declaration of Helsinki (2013).

## 3. Results

The first study goal was to analyse the relationship between the development of burnout syndrome and certain sociodemographic factors associated with it, namely age, sex, marital status, children, employment, faculty, degree subject and academic year.

The univariate analyses performed revealed a statistically significant correlation between age and EE (*r* = −0.11, *p* = 0.03), with a higher level of emotional exhaustion among younger students. The correlations with the remaining dimensions were not significant: −0.03 for D and 0.02 for PA.

Although there were no significant differences in EE between male and female students ($\bar{X}$_M_ = 15.41; $\bar{X}$_F_ = 15.03; *t*_(429)_ = −0.60; *p* = 0.551), statistically significant differences were observed in D ($\bar{X}$_M_ = 13.93; $\bar{X}$_F_ = 12.13; *t*_(420)_ = −3.10; *p* = 0.002; *d* = 0.31) and in PA ($\bar{X}$_M_ = 24.36; $\bar{X}$_F_ = 27.41; *t*_(419)_ = 4.79; *p* < 0.001; *d* = 0.48), showing that the female students presented less D and higher PA than their male counterparts.

There were no significant differences according to the students’ marital status, single or in partnership (S vs. P) in the dimensions of burnout: EE ($\bar{X}$_S_ = 15.20; $\bar{X}$_P_ = 14.47; *t*_(429)_ = 0.50; *p* = 0.619). D ($\bar{X}$_S_ = 12.82; $\bar{X}$_P_ = 12.25; *t*_(420)_ = 0.50; *p* = 0.667); PA ($\bar{X}$_S_ = 26.22; $\bar{X}$_P_ = 27.31; *t*_(419)_ = 0.72; *p* = 0.474). Neither were significant differences observed with respect to the students’ employment, as work (W) vs. no work (NW): EE ($\bar{X}$_NW_ = 15.05; $\bar{X}$_W_ = 16.03; *t*_(429)_ = −1.01; *p* = 0.315). D ($\bar{X}$_NW_ = 12.62; $\bar{X}$_W_ = 14.14; *t*_(420)_ = −1.73; *p* = 0.085); PA ($\bar{X}$_NW_ = 26.24; $\bar{X}$_W_ = 26.50; *t*_(419)_ = −0.26; *p* = 0.796).

The original variable ‘Number of children’ (continuous) was modified as participants with children or without children (C vs. NC), because the majority of the sample population had no children. No statistically significant differences were found: EE ($\bar{X}$_NC_ = 15.27; $\bar{X}$_C_ = 13.11; *t*_(429)_ = 1.41; *p* = 0.160); D ($\bar{X}$_NC_ = 12.82; $\bar{X}$_C_ = 12.35; *t*_(420)_ = 0.35; *p* = 0.725); PA ($\bar{X}$_NC_ = 26.21; $\bar{X}$_C_ = 27.47; *t*_(419)_ = −0.82; *p* = 0.410). Statistically significant differences were observed in the ‘Faculty’ variable in D (*F*_(419, 2)_ = 13.58; *p* < 0.001, *R^2^_Aj._* = 0.06), but not in EE (*F*_(428, 2)_ = 1.66; *p* = 0.192) nor PA (*F*_(418, 2)_ = 2.23; *p* = 0.103). The post hoc analyses revealed that there were significant differences between the Social Science (SS) group and the Nursing (N) group ($\bar{X}$_SS_ − $\bar{X}$_N_ = 2.38; *t*_(419)_ = 3.22; *p* = 0.004; *d* = 0.43) and the Education (E) group and the Nursing (N) group ($\bar{X}$_E_ − $\bar{X}$_N_ = 3.57; *t*_(419)_ = 5.20; *p* < 0.001; *d* = 0.63).

In the ‘Degree subject’ variable, statistically significant differences were found in EE (*F*_(423, 7)_ = 2.72; *p* = 0.009; *R^2^_Aj._* = 0.03), D (*F*_(414, 7)_ = 7.70; *p* < 0.001; *R^2^_Aj_*_._ = 0.10) and PA (*F*_(413, 7)_ = 2.23; *p* = 0.031; *R^2^_Aj._* = 0.02). The post hoc analyses highlighted the groups among which there were significant differences for each dimension. For EE, there were significant differences between the Pre-School Education (PSE) group and the Social Education (SE) group ($\bar{X}$_PSE_ − $\bar{X}$_SE_ = −4.60; *t*_(423)_ = −3.28; *p* = 0.025; *d* = 0.66) and the Primary and Physical Education (PPE) group ($\bar{X}$_PSE_ − $\bar{X}$_PPE_ = −4.77; *t*_(423)_ = −3.19; *p* = 0.025; *d* = 0.65). Thus, the PSE degree students presented less emotional exhaustion than the students in the other two groups.

In D, differences were also obtained between the Pre-School Education group and the following groups: Primary Education (PE) ($\bar{X}$_PSI_ − $\bar{X}$_PE_ = −4.06; *t*_(414)_ = −3.30; *p* = 0.023; *d* = 0.69); Primary and Physical Education ($\bar{X}$_PSE_ − $\bar{X}$_PPE_ = −4.72; *t*_(414)_ = −3.90; *p* = 0.003; *d* = 0.83); and Social Education ($\bar{X}$_PSE_ − $\bar{X}$_SE_ = −5.46; *t*_(414)_ = −4.47; *p* < 0.001; *d* = 0.98). Thus, students in the Pre-School Education degree course showed lower feelings of depersonalisation than their peers in other courses. There were also significant differences between the Nursing degree students (N) and the following groups: Primary Education ($\bar{X}$_N_ − $\bar{X}$_PE_ = −3.81; *t*_(414)_ = −3.94; *p* = 0.002; *d* = 0.68); Primary and Physical Education ($\bar{X}$_N_ − $\bar{X}$_PPE_ = −4.47; *t*_(414)_ = −4.75; *p* < 0.001; *d* = 0.83); Social Education ($\bar{X}$_N_ − $\bar{X}$_SE_ = −5.20; *t*_(414)_ = −5.45; *p* < 0.001; *d* = 0.99); and Labour Relations and Human Resources (LRHR) ($\bar{X}$_N_ − $\bar{X}$_LRHR_ = −3.81; *t*_(414)_ = −3.29; *p* = 0.023; *d* = 0.68). In this case, the Nursing students obtained lower scores in depersonalisation than the students of the other courses.

In PA, only the Pre-School Education and Social Education groups presented statistically significant differences ($\bar{X}$_PSE_ − $\bar{X}$_SE_ = 4.40; *t*_(413)_ = 3.06; *p* = 0.066; *d* = 0.71). Thus, the students in the Pre-School Education degree course presented higher levels of personal accomplishment than those in the Social Education course.

For the ‘Academic year’ variable, significant differences were only obtained for EE (*F*_(427, 3)_ = 4.01; *p* = 0.008; *R^2^_Aj._* = 0.02). According to the post hoc analyses, this significant relationship was found in the differences between the first and third-year students ($\bar{X}$_1_ − $\bar{X}$_3_ = 2.78; *t*_(427)_ = 3.36; *p* = 0.004; *d* = 0.42); thus, the first-year students were found to be more emotionally fatigued than those in their third year.

The second goal was to elaborate a risk profile with significant preceding variables. Firstly, a TSCA was performed with age, sex, faculty and academic year as predictor variables. The results obtained indicated four distinct clusters of students. The silhouette measure of cohesion and separation was 0.3, indicating ‘fair’ cluster quality. The predictor importance indicated that the variables faculty, academic year and sex were very important for the clustering solution (score range: 0.9–1.0). The composition of the clusters can be seen in Table 1. A one-way ANOVA was conducted to detect differences between the clusters. Statistically significant differences were found in PA (*F*_(415, 3)_ = 6.55; *p* < 0.001; *R^2^_Aj._* = 0.04). The students in cluster 1 had higher scores than those in cluster 3 ($\bar{X}$_1_ − $\bar{X}$_2_ = −2.56; *t*_(415)_ = −2.83; *p* = 0.025; *d* = 0.39) and in cluster 4 ($\bar{X}$_1_ − $\bar{X}$_4_ = −3.47; *t*_(415)_ = −4.14; *p* < 0.001; *d* = 0.57). Likewise, the students in cluster 2 had higher scores than those in cluster 4 ($\bar{X}$_2_ − $\bar{X}$_4_ = −2.41; *t*_(415)_ = −2.67; *p* = 0.039; *d* = 0.42).

Since differences between general clusters were not found in EE and D, and the effect size was low in PA, three additional TSCA were conducted separately, specifically for those students with high values in EE and D (≥percentile 75), and low values in PA (≤percentile 25). The reason for doing this was to reveal the clusters in which students presented some symptoms of burnout. The predictors in these models were the same as for the first general model and the number of clusters was set to four. In the three specific models, the silhouette measure was equal to or higher than 0.30 (fair). The composition of the clusters for each dimension is shown in Table 2.

Finally, multiple linear regression models were performed, using the backward stepwise method. As shown in Table 3, in EE the model was statistically significant (*F*_(387, 6)_ = 3.53; *p* = 0.002; *R^2^_Aj._* = 0.04), with academic year and degree subject being the significant variables. First-year students had more EE than third-year students, and the SE and PPE groups had higher scores than the PSE group. Both in D (*F*_(387, 6)_ = 8.41; *p* < 0.001; *R^2^_Aj._* = 0.11) and in PA (*F*_(385, 8)_ = 5.05; *p* < 0.001; *R^2^_Aj._* = 0.08) the models were statistically significant, with sex and degree subject being the statistically significant variables. Male students had higher scores for D and lower ones for PA than female students. The PPE and SE groups presented higher scores for D than the N group, and the SE group obtained lower scores for PA than the PSE group.

## 4. Discussion

This study analyses the relationship between the development of the academic burnout syndrome and the following sociodemographic factors: age, gender, marital status, children, employment status, faculty, degree subject and academic year. The predictors obtained were combined in order to elaborate a risk profile of academic burnout.

According to the results obtained, age, marital status, number of children and employment are not associated with the appearance of this syndrome among the students. This result is in line with previous findings regarding students’ age [23] and employment status [21]. On the other hand, sex, degree subject, faculty and academic year do seem to bear some relation to the presence of the syndrome, which corroborates earlier results in this vein [3,26,33,34]. Regarding the variable ´Sex´, male students suffered more depersonalisation and had a lower level of personal accomplishment. We speculate that differences in depersonalisation may be related to differences in the use of coping resources to address the problems that arise during academic life. Depersonalisation is characterised by difficulties in preserving interpersonal relationships, i.e., with classmates and teachers, in the case in question. The coping style of female students appears to be more focused on emotions and empathy than among their male counterparts [35]. This difference might explain the higher level of depersonalisation observed in the male students.

Regarding the ‘University degree subject’ variable, significant differences were obtained in all three dimensions. Students in the Primary Education course were more fatigued, presented more cynicism and felt a lower level of personal accomplishment than those studying Pre-school Education and Nursing. The students in the Social Education course experienced a greater feeling of depersonalisation than those in Nursing, and a lower sense of personal accomplishment than those studying Pre-school Education. Thus, it seems that the students most likely to develop burnout syndrome are those studying for degrees in Primary Education and Social Education.

The first-year students tended to be more emotionally exhausted than their peers in later years, although the levels of fatigue were not especially high and the differences only moderate. This tendency was also observed in the significant, but low, correlation between the students’ age and the degree of emotional exhaustion suffered. In this sense, younger students expressed more emotional exhaustion than older students.

A risk profile of the onset of burnout syndrome was elaborated, based on cluster analysis and regression models. In the first of these respects, the results for the students with high emotional exhaustion, high depersonalisation and low personal accomplishment indicated that sex, faculty and academic year are the most important variables with respect to impact on burnout. Taking into account the cluster characteristics, a profile was elaborated in each dimension of burnout syndrome. Students with high emotional exhaustion were typically first-year males in the Education faculty and fourth-year males in the Social Science faculty. Students with high depersonalisation were typically females and third-year males in the Education faculty and first-, second- and fourth-year males in the Social Science faculty. Students with low personal accomplishment were typically those enrolled in the Education faculty, plus fourth-year males in the Nursing faculty and males in their first, second and fourth academic year. 

The regression models showed that the highest levels of emotional exhaustion corresponded to the first-year students and those in the Primary Education and Social Education courses. The male students and those in the Primary Education and Social Education courses presented higher levels of depersonalisation and lower levels of personal accomplishment. The effect sizes in the multivariate analyses were low, and most of those in the univariate analysis were of low or intermediate magnitude.

Application of the above analytic strategies produced a risk profile of sociodemographic factors relevant to the onset of academic burnout. The variables sex, faculty and academic year were related to high levels of burnout in the students considered. Specifically, male students in the Education faculty were found to be at greater risk of developing burnout syndrome than their counterparts, since they experienced high levels of exhaustion and depersonalisation and low levels of personal accomplishment. Furthermore, first and fourth-year students had higher levels of emotional exhaustion, presenting a quadratic trend. Hence, the risk profile of sociodemographic factors for the onset of academic burnout is characterised mainly by first and fourth-year male students in the Education faculty. Nevertheless, the impact of these variables is only slight in the regression models, which suggests there may be other relevant variables, such as psychological or educational factors. Similar results have been obtained in previous studies of student populations [3,26,33,34].

The present study has some limitations. First, it was conducted by convenience sampling; sampling by quotas, that is, maintaining the proportions of students from the three faculties of the campus, would alleviate this limitation. Second, the reliability of the depersonalisation dimension was somewhat lower than the value commonly accepted [36]. Although this result has been obtained in a previous study [37], the results in this respect should be interpreted with caution. In future research, it would be advisable to assess larger sample sizes of the same population and to perform replication studies in other universities. In addition, future studies should explore psychological factors (e.g., personality, self-efficacy, coping style) and the educational services provided (e.g., infrastructure and materials and academic services), which may be associated with academic burnout syndrome [13,14].

## 5. Conclusions

In this study, a risk profile was obtained of sociodemographic factors relevant to the onset of academic burnout. Those most likely to experience this condition were first- and fourth-year male students in the Education faculty. University policy makers and researchers in the field of academic burnout should take these factors into account when developing intervention and prevention programmes. In view of these findings, we believe it is essential to design effective programmes to combat the academic burnout syndrome. In parallel with the above, future studies should seek a deeper understanding of the educational (e.g., welcome and vocational guidance services) and psychological factors (e. g., coping strategies) involved in order to make an accurate assessment of academic burnout syndrome.

## Figures and Tables

**Table 1:** Cluster analysis profile with the full sample (*n* = 443).

Variables	Cluster 1 (*n* = 105; 23.6%)	Cluster 2 (*n* = 79; 17.8%)	Cluster 3 (*n* = 103; 23.1%)	Cluster 4 (*n* = 156; 35.1%)
Age, M ± SD	21.38 ± 3.29	20.22 ± 2.72	24.50 ± 6.60	21.24 ± 3.23
Sex, female (%)	0	14.4	29.2	56.3
Academic year (%)				
First	25			
Second	24	24.8	2.3	48.8
Third	27.6	0	72.4	0
Fourth	0	0	100	0
Faculty (%)				
Social Sciences	0	59.8	40.2	0
Education	39.4			
Nursing	24.6	0	16.1	59.3
EE, M ± SD	15.45 ± 6.57	16.22 ± 6.08	13.97 ± 7.04	15.22 ± 6.25
D, M ± SD	13.86 ± 5.55	13.01 ± 5.75	12.50 ± 5.99	12.29 ± 6.07
PA, M ± SD	24.24 ± 6.30	25.30 ± 6.72		

**Table 2:** Cluster analysis profile in students with high levels of burnout.

Dimensions/Variables	Cluster 1	Cluster 2	Cluster 3	Cluster 4
**High EE**	(*n* = 15; 12.9%)	(*n* = 29; 25%)	(*n* = 47; 40.5%)	(*n* = 25; 21.6%)
Age, M ± SD	19.53 ± 1.64	23.79 ± 8.19	21.19 ± 2.13	21.36 ± 4.66
Sex, female (%)	0	27.1	37.1	35.7
Academic year (%)				
First	25.9	31	0	43.1
Second	0	3.8	96.2	0
Third	0	0	100	0
Fourth	0	100	0	0
Faculty (%)				
Social Sciences	0	65.6	34.4	0
Education	25.4	1.7	52.5	20.3
Nursing	0	28	20	52
**High D**	(*n* = 25; 23%)	(*n* = 29; 26.9%)	(*n* = 15; 13.9%)	(*n* = 39; 36.1%)
Age, M ± SD	21.09 ± 1.64	24.14 ± 8.34	22.13 ± 2.99	21.08 ± 4.78
Sex, female (%)	0	8.5	25.4	66.1
Academic year (%)				
First	12.8	43.6	7.7	35.9
Second	19.4	27.8	8.3	44.4
Third	56.5	0	8.7	34.8
Fourth	0	20	70	10
Faculty (%)				
Social Sciences	16.7	44.4	38.9	0
Education	33.3	0	0	66.7
Nursing	0	86.7	6.7	6.7
**Low PA**	(*n* = 31; 26.1%)	(*n* = 33; 27.7%)	(*n* = 33; 27.7%)	(*n* = 22; 28.5%)
Age, M ± SD	20.68 ± 2.15	23.94 ± 6.85	21.79 ± 2.13	20.27 ± 2.14
Sex, female (%)	52.5	37.3	10.2	0
Academic year (%)				
First	29.4	27.5	0	43.1
Second	23.1	15.4	61.5	0
Third	30.4	30.4	39.1	0
Fourth	0	100	0	0
Faculty (%)				
Social Sciences	0	25	44.4	30.6
Education	51.7	1.7	28.3	18.3
Nursing	0	100	0	0

**Table 3:** Summary of the multiple linear regression model for Emotional Exhaustion, Depersonalisation and Personal Accomplishment.

Predictor	*B*	Standard Error	Beta	*t*	*p*
**Emotional Exhaustion**					
Intercept	13.04	0.56		23.21	<0.001
BAM + L vs. PSE	2.49	1.26	0.11	1.98	0.048
PE vs. PSE	1.63	1.08	0.08	1.51	0.132
SE vs. PSE	2.62	1.05	0.13	2.51	0.013
PPE vs. PSE	2.43	1.05	0.12	2.32	0.021
1 vs. 3	2.09	0.69	0.16	3.03	0.003
4 vs. 3	2.21	1.34	0.09	1.65	0.100
**Depersonalisation**					
Intercept	10.45	0.46		22.49	<0.001
Male vs. Female	1.32	0.62	0.11	2.14	0.033
BAM vs. N	1.99	0.84	0.12	2.37	0.018
LRHR vs. N	3.40	1.13	0.15	3.02	0.003
PE vs. N	3.55	0.94	0.19	3.78	<0.001
SE vs. N	4.69	0.92	0.26	5.09	<0.001
PPE vs. N	3.63	0.97	0.20	3.75	<0.001
**Personal Accomplishment**					
Intercept	28.34	0.45		62.99	<0.001
Male vs. Female	−3.28	0.65	−0.25	−5.07	<0.001
LRHR vs. PSE	−1.99	1.20	−0.08	−1.66	0.099
PE vs. PSE	−1.91	0.99	−0.09	−.192	0.055
SE vs. PSE	−3.81	0.98	−0.19	−3.89	<0.001

BAM + L = Double Degree in Business Administration and Management and Law; PSE = Degree in Pre-School Education; PE = Degree in Primary Education; SE = Degree in Social Education; PPE = Double Degree in Primary and Physical Education; 1 = First year; 3 = Third year; 4 = Fourth year. BAM = Degree in Business Administration and Management; N = Degree in Nursing; LRHR = Degree in Labour Relations and Human Resources.
